# Sexual polyploidization in plants – cytological mechanisms and molecular regulation

**DOI:** 10.1111/nph.12184

**Published:** 2013-02-20

**Authors:** Nico De Storme, Danny Geelen

**Affiliations:** Department of Plant Production, Faculty of Bioscience Engineering, University of GhentCoupure Links 653, B-9000, Gent, Belgium

**Keywords:** 2n gametes, meiotic restitution, parallel spindles, pre- and post-meiotic genome duplication, sexual polyploidization

## Abstract

In the plant kingdom, events of whole genome duplication or polyploidization are generally believed to occur via alterations of the sexual reproduction process. Thereby, diploid pollen and eggs are formed that contain the somatic number of chromosomes rather than the gametophytic number. By participating in fertilization, these so-called 2n gametes generate polyploid offspring and therefore constitute the basis for the establishment of polyploidy in plants. In addition, diplogamete formation, through meiotic restitution, is an essential component of apomixis and also serves as an important mechanism for the restoration of F1 hybrid fertility. Characterization of the cytological mechanisms and molecular factors underlying 2n gamete formation is therefore not only relevant for basic plant biology and evolution, but may also provide valuable cues for agricultural and biotechnological applications (e.g. reverse breeding, clonal seeds). Recent data have provided novel insights into the process of 2n pollen and egg formation and have revealed multiple means to the same end. Here, we summarize the cytological mechanisms and molecular regulatory networks underlying 2n gamete formation, and outline important mitotic and meiotic processes involved in the ectopic induction of sexual polyploidization.

## Contents

Summary 670

I. Introduction 670

II. General mechanisms of 2n gamete formation 671

III. Cytological processes leading to meiotic restitution 672

IV. Genetic control of 2n gamete formation 675

V. Conclusions and future directions 680

Acknowledgements 680

References 680

## I. Introduction

Polyploidy is generally defined as the possession of three or more complete copies of the nuclear chromosome set. In plants, polyploidy was discovered a century ago (Strasburger, 1910; Winkler, 1916) and is considered to be an important feature of chromosome evolution (Ramsey & Schemske, 1998). For a long time, polyploidy was viewed as an evolutionary dead end, but the widespread occurrence in natural populations (Wood *et al*., 2009) suggests that it represents a major evolutionary force driving both speciation and diversification (Otto & Whitton, 2000; Soltis *et al*., 2009). The excess of genomic material in polyploids confers a high level of genomic plasticity, providing an evolutionary advantage over their diploid complements (Hegarty & Hiscock, 2008; Leitch & Leitch, 2008; Van de Peer *et al*., 2009). In line with this, recent research has demonstrated that several plant genomes harbor evidence of past polyploidization (Wendel, 2000; Adams & Wendel, 2005). Moreover, phylogenomic analysis has shown that plant evolution is characterized by two ancient episodes of genome duplication, indicating that almost all extant seed plants are ancestrally polyploid (Jiao *et al*., 2011).

In general, polyploids are either classified as auto- or allopolyploid, largely based on the mode of origin. Autopolyploids typically originate from a polyploidization event within or between populations of a single species, whereas allopolyploids are the result of a hybridization event between different biological species (Comai, 2005). In the latter, genome duplication serves as a mechanism to restore the fertility of newly formed amphihaploid F1 hybrids (e.g. restoration of bivalent homolog interaction; Lu & Bridgen, 1997; Ramanna *et al*., 2003; Kynast *et al*., 2012), providing additional evidence that polyploidy is an important driver for plant evolution.

Whole genome duplication may occur by somatic chromosome doubling (somatic polyploidization) or sexually through gametic nonreduction (sexual polyploidization). In somatic polyploidization, the ectopic formation of polyploid tissue, through endomitosis, nuclear fusion and/or c-mitosis, eventually leads to the stable establishment of polyploidy. By contrast, in the process of sexual polyploidization, polyploids are generated by the formation and fusion of diploid gametes, that is pollen or eggs having the somatic chromosome number rather than the gametophytic number. These gametes are also called diplogametes or 2n gametes.

Up to the 1970s, polyploid plants were thought to result from somatic genome duplication. However, when Harlan & De Wet (1975) found that several plant genera produce 2n gametes, it was proposed that sexual polyploidization represents the major route for polyploid induction. During the following decades, this idea was further supported by the identification of a wide variety of plant species that produce 2n gametes, and the finding that abiotic stress, and particularly temperature, also induces 2n gamete formation (Mason *et al*., 2011; Pecrix *et al*., 2011; De Storme *et al*., 2012). As such, it is now generally accepted that polyploid plant lineages result from sexual polyploidization, for example through the functioning of 2n gametes.

## II. General mechanisms of 2n gamete formation

Several cytological mechanisms have been described that generate 2n gametes. These mechanisms can be subdivided into three developmental-specific classes: pre- and post-meiotic genome doubling and meiotic restitution.

### 1. Genome duplication vs meiotic restitution

Diploid gametes can be produced by pre-meiotic genome doubling. In this mechanism, the ectopic induction of pre-meiotic endomitosis, endoreduplication or nuclear fusion generates tetraploid meiocytes, which, on normal meiosis, eventually lead to diploid gametes. Although repeatedly described as a component of parthenogenesis and asexuality in animals (Cuellar, 1971; Heppich *et al*., 1982; Yoshikawa *et al*., 2009; Lutes *et al*., 2010; Neaves & Baumann, 2011), this mechanism has only sporadically been documented in plants. For example, in *Turnera* hybrids, male meiocytes occasionally contain double the amount of chromosomes, indicating that 2n pollen originates from pre-meiotic genome doubling (Fernandez & Neffa, 2004). Similarly, the formation of giant tetrads in Brassica hybrids and orchids was presumed to indicate pre-meiotic chromosome duplication (Teoh, 1984; Mason *et al*., 2011). Moreover, in *Chrysanthemum*, 2n pollen originates from the fusion of two adjacent pollen mother cells (PMCs) in the early stages of meiosis I (MI; Kim *et al*., 2009). The small number of reports documenting pre-meiotic genome duplication suggests that this mechanism of 2n gamete formation is rather rare. However, as the detection of pre-meiotic genome doubling is technically challenging, one cannot exclude the possibility that it constitutes a major pathway in natural 2n gamete formation. Indeed, for several species, the production of diploid gametes has been demonstrated, but the underlying cytological mechanism is, as yet, unknown (De Haan *et al*., 1992; Chen *et al*., 2008; Seal *et al*., 2012).

Alternatively, 2n gametes can result from post-meiotic genome duplication, also termed post-meiotic restitution (PMR) or post-meiotic doubling (PMD). In this mechanism, meiotically formed haploid spores undergo an extra round of genome duplication, and consequently yield fully homozygous 2n gametes. Similar to pre-meiotic genome duplication, PMD has only rarely been documented in plants. In a specific potato hybrid, marker-assisted genotyping demonstrated that the 2n eggs are fully homozygous and thus originate from PMD (Bastiaanssen *et al*., 1998). Diploidization of egg cells has also been observed in interspecific hybrids of sugarcane and in *Rubus laciniatus* (Dowrick, 1966; Ramanna & Jacobsen, 2003), indicating that PMD constitutes a naturally occurring mechanism for 2n gamete formation.

In most cases, however, 2n gametes result from a restitution of the meiotic cell cycle (Bretagnolle & Thompson, 1995). In this process, meiotic cell division is converted into a mitosis-like nonreductional process, generating dyads (and triads) instead of the normal tetrads at the end of meiosis II (MII). This phenomenon is referred to as ‘meiotic restitution’ or ‘meiotic nonreduction’, and spores generated by this process are termed ‘unreduced gametes’. In contrast with pre- and post-meiotic genome doubling, meiotic restitution has repeatedly been documented in several species and under different stress conditions (Pecrix *et al*., 2011; De Storme *et al*., 2012), indicating that it is the predominant mechanism of 2n gamete formation in plants.

### 2. First division restitution (FDR) vs second division restitution (SDR)

**Fig. 1:**
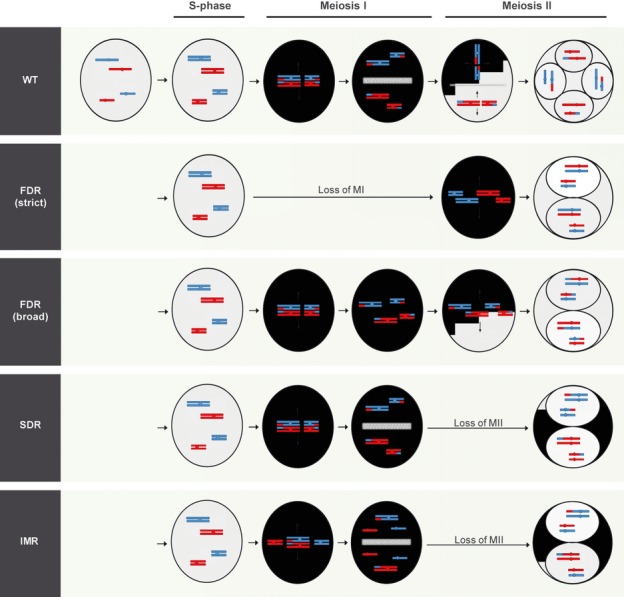
Types of 2n gamete-forming mechanism based on the genotypic outcome. Classification of 2n gamete-forming cytological mechanisms based on the genetic make-up of the generated diplogametes and graphical representation of the equivalent meiotic restitution, as observed in simultaneous-type meiotic cell division. For simplicity, the meiotic cell is diploid and only contains two chromosomes that are fully heterozygous (e.g. blue and red chromosomes obtained from genetically different parents). FDR, first division restitution; IMR, indeterminate meiotic restitution; MI, meiosis I; MII, meiosis II; SDR, second division restitution.

In addition to a cytological classification, 2n gamete-forming processes can also be subdivided according to the genetic outcome of the resulting gametes. Indeed, as 2n gametes originating from a heterozygous plant can confer high levels of heterozygosity or homozygosity, the underlying mechanism can be classified as FDR or SDR, respectively (Fig. 1; Brownfield & Köhler, 2011).

In FDR-type mechanisms, by definition, 2n spores are genetically equivalent to the gametes formed by an omission of MI. As, in this process, recombination is abolished and chromosomes undergo directly an equational division, the meiotic cell division is completely converted into a mitotic division, generating 2n gametes that are genotypically identical to the parent. Thus, in the strict sense, FDR fully retains parental heterozygosity and epistasis. Strikingly, in some types of FDR, parental heterozygosity is not retained through the loss of MI, but through other cytological alterations (e.g. parallel spindles (ps)). As in these cases, MI is not omitted, the resulting 2n gametes have undergone recombination and thus only partially retain parental heterozygosity, more specifically in the genomic regions from the centromere to the first cross-over (CO; Ramanna & Jacobsen, 2003).

In SDR mechanisms, however, the genotypic constitution of the 2n gametes is equivalent to those formed by a complete loss of MII. As, in this process, MI proceeds as normal, with correct pairing and recombination, the resulting 2n gametes are always homozygous from the centromere to the first CO, but retain parental heterozygosity at the telomeric side (Ramanna & Jacobsen, 2003). As a result, SDR-type 2n gametes confer a reduced level of heterozygosity and show a substantial loss of parental epistasis (Peloquin *et al*., 2008).

Based on these definitions, the differentiation between FDR and SDR basically relies on the genotypic constitution of the 2n gametes: spores that retain parental heterozygosity at the centromeres point towards FDR, whereas a loss of centromeric heterozygosity indicates SDR (Bretagnolle & Thompson, 1995). The decision as to whether a particular 2n gamete-forming process occurs by FDR or SDR can therefore be determined not only by the genotypic characterization of the gametes, but also from cytological examination (Douches & Quiros, 1988; Ramanna & Jacobsen, 2003). Cytological alterations that allow for the co-localization of sister chromosomes in the resulting 2n gamete are considered as SDR, whereas mechanisms that result in the co-localization of nonsisters indicate FDR.

In addition to FDR and SDR, a third class of 2n gamete-forming process has been suggested, namely indeterminate meiotic restitution (IMR). Using genomic *in situ* hybridization (GISH), Lim *et al*. (2001) found that interspecific *Lilium* hybrids produce PMCs containing a mix of univalents and bivalents that divide equationally and reductionally, respectively, generating 2n gametes that only partially retain parental heterozygosity at the centromere (Zhou *et al*., 2008). As the resulting 2n gametes varied between FDR and SDR, IMR was introduced as an alternative term to define cytological processes that simultaneously display SDR- and FDR-type restitution in one single meiocyte (Ramanna & Jacobsen, 2003).

## III. Cytological processes leading to meiotic restitution

Restitution of the meiotic cell cycle can be achieved by a plethora of cytological mechanisms, which are generally subdivided into three main classes: (1) alterations in spindle biogenesis and polarity; (2) cytokinetic defects; and (3) complete omission of a meiotic cell division.

### 1. Alterations in meiotic spindle morphology and orientation

In several plant species, 2n gametes are formed by the absence or malformation of the spindle during metaphase I or II. Failure of spindle biogenesis in MI inherently impairs the segregation of chromosomes and generally leads to a complete loss of reductional division. Subsequent equational division consequently mimics a mitotic division and generates FDR-type 2n nuclei (Wagenaar, 1968). However, as MI spindle defects do not impair recombination, the resulting 2n gametes are not fully clonal, but confer some level of homozygosity. In the case of a total lack of recombination, loss of MI spindle formation completely converts meiosis into a mitotic division, generating 2n gametes that are genetically identical to the parent. This mechanism spontaneously occurs in newly formed amphihaploid hybrids, for example in interspecific cereal hybrids (Xu & Joppa, 2000; Jauhar, 2007). In these plants, the two different parental chromosome sets do not pair, and consequently form univalents instead of bivalents. As a result, metaphase I is delayed and MI spindle biogenesis is altered, leading to a restitution of MI (Zhang *et al*., 2008).

In contrast with spindle alterations in MI, which are only rarely observed in plants, aberrations in MII spindle biogenesis more frequently lead to 2n gametes. Thereby, co-orientation of second division spindles, generally referred to as parallel spindles (ps), is the most common mechanism inducing 2n gametes (Veilleux, 1985; Bretagnolle & Thompson, 1995). This type of anomaly only occurs in PMCs of species that display a simultaneous-type cytokinesis (Furness & Rudall, 2000; Furness *et al*., 2002; Albert *et al*., 2011). In contrast with the successive-type of PMC, in which a single cell plate is installed after each meiotic division, the simultaneous type does not perform cytokinesis after MI, but instead forms a ‘double’ cell wall at the end of MII. In this process, the two chromosome sets generated in MI remain in a common cytoplasm during MII and, to prevent potential interference, MII spindles are perpendicularly organized, tightly controlling the spatial organization of the two chromosome sets and constituting the basis for the tetrahedral configuration of the haploid nuclei at the end of MII (d'Erfurth *et al*., 2008).

**Fig. 2:**
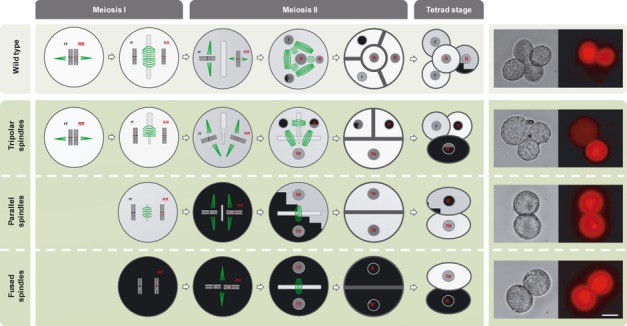
Cytological mechanism of tripolar, parallel and fused spindles, and the associated formation of first division restitution (FDR)-type 2n gametes. Graphical representation of the process of meiotic nonreduction through aberrations in meiosis II (MII) spindle orientation, and the presumed link with alterations in meiosis I (MI)-to-MII interzonal microtubule array formation. The segregation of a heterozygous centromeric marker (Rr) is represented by the black (r) and red (R) lines. Microtubular structures are represented by green fluorescent figure arrays. Additional tetrad figures in the Arabidopsis *parallel spindle* (*ps*) mutant *jas*, harboring a heterozygous centromere-linked pollen fluorescence marker (e.g. FTL 1323; dsRed2; Francis *et al*., 2007), indicate that ps-generated diploid spores in restituted dyads and triads maintain parental heterozygosity in genomic regions close to the centromere. Bar, 20 μm.

In ps, the orientation of MII spindles is altered and converted from a perpendicular to a parallel configuration (Andreuzza & Siddiqi, 2008). As, thereby, the normal number of four MII poles is reduced to two, ps typically generate dyads that contain two diploid spores instead of the normal tetrads with four haploid spores (Genualdo *et al*., 1998). In ps-type meiotic restitution, chromatids of previously separated chromosomes are rejoined at the end of MII, resulting in FDR-type 2n gametes (d'Erfurth *et al*., 2008; De Storme & Geelen, 2011). In most species, the frequency of ps and subsequent dyad formation is consistent (Lopez-Lavalle & Orjeda, 2002); however, in some reports, a clear discrepancy between the spindle defect and meiotic restitution was observed (Carputo *et al*., 1995; Barone *et al*., 1997). In these plants, ps do not always result in restituted dyads, but instead lead to normal tetrads. A putative explanation was provided by Zhang & Kang (2010), by demonstrating that some ps PMCs in poplar (*Populus*) still produce cytokinetic radial microtubule arrays (RMAs) in between all four nuclei and generate normal tetrads, whereas other ps PMCs display only two or three RMA domains, yielding dyads and triads, respectively. These findings demonstrate that ps-mediated meiotic restitution essentially constitutes alterations in the spatial positioning of the haploid nuclei and associated aberrations in cell plate formation at the end of MII (Fig. 2).

Other MII spindle defects leading to meiotic nonreduction are tripolar (tps) and fused (fs) spindles (Rim & Beuselinck, 1996). In tps, unilateral defects in MII spindle orientation lead to a rejoining of chromatids at one pole and normal separation at the other pole, producing triads with one diploid and two haploid spores. In fs-type meiotic restitution, the complete fusion of MII spindles converts the double equational division into a single mitotic division. As these processes are generally observed together with ps, it is suggested that they are caused by a similar cytological defect and that their level of appearance only depends on the severity of the underlying defect (Veilleux & Lauer, 1981). Male-specific meiotic restitution through ps, tps and fs has been documented in a large set of plant species (Souter *et al*., 1980; Parrott & Smith, 1984; Teoh, 1984; Conicella *et al*., 1991; Rim & Beuselinck, 1996; Genualdo *et al*., 1998; Lopez-Lavalle & Orjeda, 2002; Crespel *et al*., 2006; Zhang & Kang, 2010), and is therefore considered to be the predominant route for 2n gamete formation in plants. However, despite the high prevalence, the exact cytological anomaly underlying this type of meiotic restitution is still unknown. Our current hypothesis is that the shift from a bipolar to a quadripolar spindle orientation in MII depends on the physical separation of the two daughter nuclei after MI, for example, through the establishment of the primary interzonal microtubule array (IMA; Conicella *et al*., 2003), and that defects in IMA and associated organelle deposition may lead to alterations in MII spindle polarity. In support of this hypothesis, Arabidopsis ps mutant PMCs (e.g. *jas* and *atps1*) often lack the typical interkinetic organelle band and frequently exhibit fused daughter nuclei at the start of MII (d'Erfurth *et al*., 2008; De Storme & Geelen, 2011). From this perspective, a complete loss of IMA would lead to fs, whereas partial or unipolar loss would lead to ps and tps, respectively (Fig. 2).

### 2. Defects in meiotic cell plate formation

A second class of meiotic restitution is characterized by defects in (post-)meiotic cytokinesis. Two main mechanisms have been reported: (1) a general failure of cytokinesis; and (2) precocious cytokinesis. In several plant species, partial or complete loss of cell plate formation generates bi- and polynuclear spores which, on nuclear fusion, eventually develop into di- and polyploid gametes (Peloquin *et al*., 1999; Boldrini *et al*., 2006; Gallo *et al*., 2007). Although mostly documented in male sporogenesis, diplogamete formation, through defects in meiotic cytokinesis, also occurs in megasporogenesis, for example in alfalfa (Tavoletti *et al*., 1990; Mariani *et al*., 1993) and potato (Werner & Peloquin, 1990). In meiocytes showing a successive type of cytokinesis, that is, in female meiocytes or in male meiocytes of monocotyledonous plants, loss of cell plate formation can occur after MI or after MII, producing FDR or SDR 2n gametes, respectively (Pfeiffer & Bingham, 1983; Pagliarini *et al*., 1999; Boldrini *et al*., 2006). By contrast, in simultaneous-type PMCs (e.g. in all dicotyledonous plants), loss of cell plate formation always occurs at the end of MII and, depending on the severity, generates di- or polyploid pollen. Specifically considering 2n gamete formation, alterations in post-meiotic cytokinesis occur randomly and produce a mix of SDR- and FDR-type gametes (Tavoletti *et al*., 1990). In the case of a complete loss of meiotic cytokinesis, tetranuclear spores are formed, which eventually develop into tetraploid gametes (Pfeiffer & Bingham, 1983; Spielman *et al*., 1997; Gallo *et al*., 2007). Loss of meiotic cytokinesis not only occurs on mutation, but has also repeatedly been documented under natural conditions (Bielig *et al*., 2003; De Storme *et al*., 2012), indicating that it may constitute an important mechanism underlying sexual polyploidization in plants.

Another meiotic cytokinesis defect generating 2n gametes is the premature installation of a cell plate in meiocytes that have a simultaneous-type cytokinesis. In this process, MI is followed directly by cytokinesis and MII does not occur, except for the fact that the chromatids fall apart. As a result, dyads containing SDR 2n spores are formed (Peloquin *et al*., 1999; Zhang *et al*., 2009). This mechanism was first described in potato (*premature cytokinesis pc* mutant; Mok & Peloquin, 1975a,b) and was later reported in several wild-type species, including poplar and wheat (Watanabe & Peloquin, 1993; Shamina *et al*., 2009; Zhang *et al*., 2009). In some species and hybrids (e.g. *Triticum*–*Aegilops*), premature cytokinesis occurs even earlier in meiocyte development, for example during MI, generating asymmetrical dyads with one empty and one diploid cell (Xu & Dong, 1992). Subsequent progression through MII then yields a dyad with two FDR 2n gametes. Altogether, these findings indicate that alterations in meiotic cytokinesis, depending on the timing, result in either FDR- or SDR-type 2n gametes.

### 3. Complete loss of first or second meiotic division

A complete loss of MII has been documented in several species, particularly in female sporogenesis, and generates 2n spores that largely lose parental heterozygosity (Stelly & Peloquin, 1986; Conicella *et al*., 1991; Jongedijk *et al*., 1991; Ortiz & Peloquin, 1991; Werner & Peloquin, 1991; Tavoletti, 1994; Lim *et al*., 2004). However, as MI recombination still allows for genomic exchange between the parental homologs, 2n gametes resulting from the loss of MII are homozygous towards the centromeres (SDR), but retain heterozygosity towards the telomeres.

In the case of a complete loss of MI, however, homolog duplex formation is omitted, and the meiotic cell division is fully converted into a mitotic division, disjoining chromatids instead of chromosomes. As chromatids are equally separated, the resulting dyads always contain 2n spores that are genetically identical to the parent (FDR *sensu stricto*). This process is often referred to as apomeiosis and is an essential part of the diplosporous type of gametophytic apomixis (Ozias-Akins & van Dijk, 2007; d'Erfurth *et al*., 2009). Indeed, the formation of 2n gametes, and particularly 2n eggs, through the loss of MI has frequently been documented in apomictic plant species, such as Taraxacum and Arabis (Naumova *et al*., 2001; Vijverberg *et al*., 2010). However, in sexually reproducing species, such as potato and alfalfa, loss of MI has also occasionally been observed in male and female sporogenesis (Islam & Shepherd, 1980; Werner & Peloquin, 1990; Conicella *et al*., 1991; Tavoletti, 1994; Barcaccia *et al*., 1997). In these instances, however, apomeiosis was either induced by (a) mutation(s) or indirectly by desynapsis or (amphi)haploidy (Islam & Shepherd, 1980; Ramanna, 1983; Jongedijk *et al*., 1991). Univalent-induced meiotic restitution is also referred to as pseudohomotypic division, and often occurs in intergeneric F1 hybrids as a way to circumvent F1 sterility by inducing allopolyploidy (Islam & Shepherd, 1980; Zhang *et al*., 2007, 2008; Silkova *et al*., 2009; Shamina & Shatskaya, 2011). Moreover, based on the high frequency of polyploid hybrids observed in nature and agronomy, this pathway is thought to be the predominant mechanism underlying allopolyploid plant formation (Jauhar, 2007). Despite its evolutionary relevance, however, little is known about the cytological mechanism underlying haploid-induced loss of MI. Silkova *et al*. (2003) suggested that univalent-induced meiotic restitution results from alterations in kinetochore organization (bipolar–monopolar) and chromosome condensation, inducing a mitotic-like division of half-bivalents. Moreover, studies in apomictic species have suggested that apomeiosis and the associated loss of MI are closely associated with or induced by the presence of mini- or B-chromosomes (Ozias-Akins *et al*., 1998; Sharbel *et al*., 2004, 2005). However, as no direct link has been found, more research is needed to elucidate the putative role of B-chromosomes in clonal gamete formation (Ozias-Akins, 2006).

Although most plant genera only show one type of meiotic restitution, two or multiple 2n gamete-inducing mechanisms may occur simultaneously in one species or even in one individual plant (Werner & Peloquin, 1991). In *Medicago sativa* ssp. *Falcata*, for example, megaspore mother cells (MMCs) exhibiting both loss of MI and MII occur, generating FDR and SDR 2n eggs, respectively (Tavoletti, 1994). Similarly, in *Hierochloë odorata*, PMCs show restitution of MI or MII, or sometimes both, and consequently generate pollen with varying gametophytic ploidy levels (Ferris *et al*., 1991). Thus, by combining several types of meiotic restitution, plants can generate a large variety of gametes, differing in both genetic make-up and ploidy.

## IV. Genetic control of 2n gamete formation

Heritability studies have demonstrated that recurrent selection for the 2n gamete phenotype in several plant species increases significantly its frequency of occurrence (Calderini & Mariani, 1997). In *Medicago sativa*, for example, heritability for 2n pollen and egg production amounted to 39% and 60%, respectively, over two cycles of recurrent selection (Tavoletti *et al*., 1991). Similarly, in tetraploid *Trifolium pratense*, 2n pollen frequency increased from 0.04% to 47.38% in three recurrent selection steps (Parrott & Smith, 1986). These findings strongly suggest that 2n gamete production is genetically controlled, and support the idea that the underlying cytological anomalies are regulated by a monogenic allele (Bretagnolle & Thompson, 1995; Ortiz, 1997). This assumption was confirmed by the genetic analysis of several crop mutants, which mostly displayed monogenic recessive inheritance (Rhoades & Dempsey, 1966; McCoy, 1982; Jongedijk & Ramanna, 1988; Werner & Peloquin, 1990; Ortiz & Peloquin, 1991; Carputo *et al*., 2003). In *Solanum tuberosum*, for example, all three mechanisms of 2n pollen formation (ps, pc-1 and pc-2) are controlled by a single recessive mutation (Mok & Peloquin, 1975a). Moreover, most alleles conferring sexual polyploidization appear to be highly sex specific, indicating that diplogamete formation in male and female sporogenesis is largely uncoupled (Bretagnolle & Thompson, 1995).

### 1. Molecular control of MII spindle orientation

The most common mechanism generating 2n gametes in plants is through alterations in spindle biogenesis and, particularly, by co-orientation of MII spindles. In male meiosis, the perpendicular orientation of MII division planes is essential for the physical separation of the haploid spores. However, despite the biological relevance, little is yet known about the underlying molecular mechanism(s).

The first protein involved in this process has been identified recently. Using reverse genetics, d'Erfurth *et al*. (2008) identified AtPS1 (*Arabidopsis thaliana* PARALLEL SPINDLES 1) as an important regulator of MII spindle orientation. Cytological analysis demonstrated that *atps1* PMCs exhibit severe alterations in MII spindle polarity, typically displaying ps and tps instead of the normal perpendicular spindles (Andreuzza & Siddiqi, 2008). As a result, loss of AtPS1 induces a restitution of male meiosis, generating dyads and triads that contain diploid spores (up to 65%). Female meiosis, however, is not affected. Consistent with ps in male MII, *atps1* 2n spores largely retain parental heterozygosity towards the centromeres, indicative of FDR-type meiotic restitution (d'Erfurth *et al*., 2008).

*AtPS1* encodes a plant-specific protein of 1477 amino acids with two highly conserved domains: an N-terminal Forkhead Associated (FHA) and a C-terminal PINc domain (d'Erfurth *et al*., 2008). FHA domains are well-known phosphopeptide recognition motifs that enable protein–protein interactions and are found in a diverse range of proteins that regulate intracellular signal transduction, cell cycle control, DNA repair and protein degradation (Li *et al*., 2000). PINc domains have predicted RNA-binding properties and are generally found in proteins involved in RNA processing and Nonsense-Mediated RNA Decay (NMD). As such, AtPS1 has been speculated to play a regulatory role in the quadripolar orientation of MII spindles, probably via NMD or RNA processing (d'Erfurth *et al*., 2008). However, as no targets have been identified to date, more research is needed to elucidate the putative involvement of RNA processing in MII polarity establishment. *In silico* genome analysis has demonstrated that *PS1*-like genes are present throughout the whole Viridaeplantae clade, from mosses to higher plants, where they generally occur as singletons (Cigliano *et al*., 2011).

Another protein involved in the regulation of MII spindle orientation is JASON (JAS). Similar to AtPS1, functional loss of JAS also induces ps and tps in male MII, generating dyads and triads that contain FDR-type 2n pollen (De Storme & Geelen, 2011). Originally identified in a screen for altered endosperm *PHE1* (*PHERES1*) expression, *JAS* encodes an unknown protein that does not contain any domain of described function (Erilova *et al*., 2009). Although no homologs were found in animal or other kingdoms, JAS shows conservation throughout the plant kingdom, and contains a highly conserved domain of unknown function at the C-terminus. Despite the lack of predicted functionality, expression data have demonstrated that JAS positively regulates the *AtPS1* transcript level in meiotic flower buds, indicating that JAS and AtPS1 form a mini-network that regulates MII spindle orientation (De Storme & Geelen, 2011). However, the exact mechanism by which JAS regulates AtPS1 transcript levels and the downstream action of this regulatory network is not yet known.

Similar to dicotyledonous plants, animal systems also show a perpendicular MII spindle organization. In mouse oocytes, for example, MII is characterized by the exclusion of one set of chromosomes into the polar body, whereas the other set remains included in the ooplasm, forming a spindle parallel to the cortex. On fertilization, this spindle rotates to a perpendicular polarity, strongly phenocopying the MII spindle orientation in plant PMCs. Genetic studies in animals have revealed several molecular components involved in MII spindle rotation, including microtubule- (MT), microfilament- (MF) and cytoskeleton-related proteins (kinesins, formins and myosins; Ai *et al*., 2009). Moreover, studies in *Caenorhabditis elegans* have demonstrated that MII spindle rotation requires the accumulation of dynein at the poles and that cyclin-dependent kinase-1 (CDK-1) blocks this process by inhibiting the association of dynein with MTs (Ellefson & McNally, 2011). Thus, inhibition of CDK-1 by activation of the anaphase-promoting complex (APC) in MII promotes the perpendicular rotation of the spindle, allowing meiocytes to finalize meiosis.

Although little is known about the molecular factors controlling MII spindle orientation in plants, similar cell cycle-regulated mechanisms could be involved. This idea is supported by the recent identification of the Arabidopsis type II formin FORMIN14 (AFH14). As AFH14 localizes to meiotic spindles and *afh14* PMCs frequently display ps, AFH14 is thought to play an important role in the orientation of MII spindles in male meiosis (Li *et al*., 2010). Cytological examination has demonstrated that AFH14 functions as a linking protein between MTs and MFs, and thus plays an important role in the regulation of cytoskeletal dynamics and organization. Hence, MII spindle organization in simultaneous-type PMCs is spatially (and maybe temporally) controlled by reciprocal interactions between cytoskeletal MT and MF arrays.

### 2. Genes involved in the progression of MII

Meiosis differs from mitosis in that it has two successive chromosome divisions without an intervening S-phase (Kishimoto, 2003). As it is generally believed that both processes are regulated by the same cell cycle machinery, meiosis is thought to be controlled by an altered programming of mitotic cell cycle regulators. For both MI and MII, entry into M-phase depends on high CDK activity, whereas exit from anaphase requires loss of activity. Hence, depletion of CDK activity during meiosis needs to be tightly coordinated, so that the remaining CDK level at the end of MI enables exit from MI, but still allows initiation of MII (Furuno *et al*., 1994; Iwabuchi *et al*., 2000; Stern, 2003; Marston & Amon, 2004; Futcher, 2008).

In Arabidopsis, Dissmeyer *et al*. (2007) demonstrated that the loss of CDKA;1 function in the weak *cdka;1* allele induces severe alterations in meiotic cell cycle progression, with premature cytokinesis following MI and the production of unbalanced MII products. The formation of 2n gametes, however, was not reported. CDK activity is controlled by a set of binding proteins that either promote or inhibit its activity (Inze & De Veylder, 2006). Cyclins are well-known CDK activators and promote both the mitotic and the meiotic cell cycle (Malapeira *et al*., 2005). Although Arabidopsis contains at least 50 cyclin-like proteins (Wang *et al*., 2004), only two have yet been found to play a role in meiosis: SDS (SOLO DANCERS) and TAM (TARDY ASYNCHRONOUS MEIOSIS)/CYCA1;2.

Defects in SDS induce severe losses in synapsis and bivalent formation in both male and female meiosis. As a result, *sds* sporogenesis generally produces polyads that contain aneuploid spores (De Muyt *et al*., 2009). Interestingly, *sds* PMCs also generate a subset of dyads (1.1%) and triads (3.5%), indicative of meiotic restitution. These observations, together with the finding that SDS contains a C-terminal domain resembling known B2-type cyclins, suggest that SDS is a meiosis-specific B2-like cyclin that regulates prophase I and meiotic cell cycle progression (Azumi *et al*., 2002; Chang *et al*., 2009). However, as yet, little is known about the specific function of SDS in meiosis.

The second meiosis-specific cyclin, CYCA1;2 or TAM, is a member of the cyclin A family (Magnard *et al*., 2001). CYCA1;2/TAM plays an essential role in the progression of MII in both male and female sporogenesis. CYCA1;2/TAM loss-of-function mutants typically complete MI, but fail to enter MII. Consequently, *tam* generates restituted dyads (100% and *c*. 30% in male and female, respectively) that contain SDR-type 2n gametes (d'Erfurth *et al*., 2010). A more detailed analysis of TAM comes from the temperature-sensitive *tam-1* allele. Unlike other *tam* alleles, *tam-1* does not show a complete abortion of MII, but, instead, displays a significant delay in meiotic progression, with the premature formation of MI dyads that continue MII to generate normal tetrads (Magnard *et al*., 2001). Recently, Cromer *et al*. (2012) have reported that CYCA1;2/TAM forms an active complex with the cyclin-dependent kinase CDKA;1, and that the expression of a nondestructible CYCA1;2/TAM invokes the entry into a third meiotic division. Hence, TAM/CYCA1;2 is an essential regulator of meiotic cell cycle progression, promoting entry into MII and mediating exit from meiosis, most presumably through the regulation of CDKA;1.

A second protein essential for entry into MII is OSD1 (OMISSION OF SECOND DIVISION 1). Similar to TAM, loss of OSD1 function induces a complete omission of MII, generating dyads that contain SDR-type 2n gametes (100% and *c*. 85% in male and female, respectively; d'Erfurth *et al*., 2009).

OSD1, also termed GIGAS CELL1 (GIG1) or UVI4-Like (UVI4-L), and its paralog UVI4 (for UV-B-insensitive 4), are highly conserved plant-specific proteins that do not contain any domain of known function. In Arabidopsis, loss of UVI4 enhances somatic endoreduplication, indicating that UVI4 is required for maintaining cells in the mitotic state (Hase *et al*., 2006). Recently, Iwata *et al*. (2011) have demonstrated that UVI4 physically binds to FZY-RELATED (FZR), a well-known APC/C activator, and thereby inhibits the precocious activation of the APC/C ubiquitin ligase complex. Similarly, OSD1 interacts with CDC20/FZY, another activator of the APC/C complex, and mutant forms of OSD1 ectopically induce somatic endomitosis, indicating that OSD1 not only controls meiotic progression, but also regulates exit from mitosis (Iwata *et al*., 2011). Hence, OSD1 encodes a plant-specific APC/C inhibitor and plays an important role in the regulation of both mitotic and meiotic cell cycle progression.

On combining *osd1* and *tam*, female meiotic restitution was not altered, but male meiosis exhibited a more severe phenotype, namely failure to enter MI and a complete loss of meiotic cell division, leading to tetraploid spores (d'Erfurth *et al*., 2010). Although seemingly contradictory, this observation is in agreement with the general notion that PMCs require an initial level of CDK activity to initiate meiosis, similar to mitotically dividing cells (Marston & Amon, 2004; Tanaka *et al*., 2007). Indeed, as both TAM and OSD1 promote CDK activity, either directly as a cyclin or indirectly via APC/C, combinatorial loss of both proteins is thought to reduce meiotic CDK activity more severely, eventually falling below the minimum level required for MI initiation. In this model, the normal initiation of MI in female *tam1*/*osd1* meiocytes indicates that the threshold of active CDK for the initiation of meiosis is lower in MMCs relative to PMCs. In search of a molecular link between CYCA1;2/TAM and OSD1, Cromer *et al*. (2012) found that CYCA1;2/TAM-activated CDKA;1 phosphorylates OSD1 *in vitro*, indicating that CYCA1;2/TAM, OSD1 and APC/C form a functional network that regulates meiotic cell cycle progression. One hypothesis is that OSD1 modulates the activity of APC/C in a gradient-dependent manner, tightly regulating the activity of downstream cell cycle regulators (e.g. cyclins), and that CYCA1;2/TAM controls this process through phosphorylation of OSD1. Adversely, CYCA1;2/TAM may form the target of APC/C-mediated degradation, whereas OSD1 regulates this degradation in a gradient-dependent manner, allowing the successive entry in MI and MII. However, as the APC/C target in meiosis has not been identified to date, more studies are needed to fully elucidate the regulatory network underlying meiotic cell cycle progression.

### 3. Genetic factors regulating the mitosis-to-meiosis switch

In contrast with the loss of MII, which generates SDR-type 2n gametes, omission of MI converts meiosis into a mitotic division, generating clonal 2n spores (FDR *sensu stricto*). Until now, only two genes have been identified that are involved in the mitosis-to-meiosis switch, namely DYAD/SWITCH1 and AGO104 (ARGONAUTE104).

The Arabidopsis *dyad* mutant was originally identified on the basis of its female sterility. Using cytological approaches, Siddiqi *et al*. (2000) revealed that *dyad* ovules exhibit an arrested dyad configuration with two large cells instead of the fully matured embryo sac. Meiotic chromosome spreads demonstrated that *dyad* MMC chromosomes do not synapse and form univalents instead of bivalents. However, in contrast with other univalent mutants, which generally show a random chromosome segregation in MI and MII, *dyad* MMCs skipped the first reductional division and underwent directly an equational division, generating dyads that contain FDR-type 2n megaspores (Agashe *et al*., 2002). In agreement, 2n megaspores were found to fully retain parental heterozygosity, indicating that *dyad* MMCs perform mitosis instead of meiosis (Motamayor *et al*., 2000). Male meiosis, however, is unaffected and generates normal haploid spores. Although generally sterile, *dyad* occasionally produces viable seeds (*c*. 10 seeds per plant). Most of these seeds are triploid and contain an excess of maternal genome dosage, indicating that unreduced *dyad* embryo sacs are functionally able to generate seed (Ravi *et al*., 2008). A more in-depth characterization of DYAD/SWITCH1 was obtained from *swi1.2*, a DYAD/SWITCH1 allele which displays defects in both male and female meiosis. Based on observations in *swi1.2*, Mercier *et al*. (2001) found that DYAD/SWITCH1 is required in prophase I and plays an essential role in sister chromatid cohesion and recombination. However, as DYAD/SWITCH1 encodes an unknown, plant-specific protein without any domain of known function and lacking homology to any other protein, little is known about its specific function in plant sporogenesis (Mercier *et al*., 2001).

A similar meiotic-to-mitotic switch occurs in male sporogenesis of the Arabidopsis *duet* mutant. Unlike *dyad*, *duet* 2n microspores do not execute normal gametogenesis, but instead exhibit additional somatic divisions leading to bi- and tri-nuclear microspores that eventually abort. Cytological analysis revealed that *duet* PMCs display late defects in synapsis and significantly delayed metaphase I, eventually generating restituted dyads (Reddy *et al*., 2003). The exact mechanism by which these dyads are formed, however, is as yet unknown. *DUET* encodes a putative PHD-finger protein that is specifically expressed in PMCs. Although little is known about its specific function, genetic studies have shown that DUET interacts synergistically with DYAD/SWITCH1 to maintain normal meiotic cell cycle progression (Reddy *et al*., 2003).

Recently, AGO104 has been found to regulate the mitosis-to-meiosis transition in maize. Loss of AGO104 function induces the formation of 2n male and female gametes through a restitution of the meiotic cell cycle (Singh *et al*., 2011). Cytological examination demonstrated that *ago104* meiocytes show defects in prophase I chromatin condensation, spindle formation and chromosome segregation, and consequently generate dyads, triads and polyads. Moreover, *ago104* MI chromosomes display a mitotic-like peri-centromeric phosphorylation of histone H3 at serine-10 (H3ser10P) – a chromatin state which is closely linked to sister chromatid cohesion (Kaszas & Cande, 2000) – rather than the MI-specific whole chromosome labeling pattern, suggesting that *ago104* dyads result from a mitotic rather than a meiotic division. Hence, loss of AGO104 induces the formation of clonal 2n gametes (apomeiosis), an essential component of diplosporous apomixis.

**Fig. 3:**
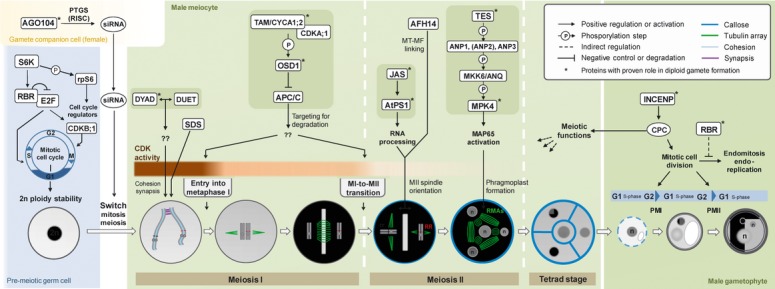
Overview of the molecular factors involved in diploid and polyploid gamete formation in plants. Graphical representation of all proteins and molecular networks controlling pre- and post-meiotic ploidy stability, meiotic genome reduction and haploid spore formation in plants. Proteins that are known to generate diploid and/or polyploid gametes on mutation are indicated by asterisks. Please see main text for definitions of abbreviations.

The maize AGO104 contains the characteristic PAZ and PIWI domains found in all ARGONAUTE proteins. AGOs and related PIWI proteins are present in plants, animals and fungi, and play an important role in epigenetic transcriptome regulation, for example through post-transcriptional gene silencing (RNA-induced silencing complex (RISC) component) and RNA interference (RNAi)-induced chromatin modification (Hutvagner & Simard, 2008). In this process, both motifs play an essential role: the PAZ domain specifically binds to RNA, whereas the PIWI domain has an RNaseH-like endonuclease activity (Cerutti *et al*., 2000; Ma *et al*., 2004; Song *et al*., 2004). In line with this, AGO104 is required for the methylation of non-CG sites in centromeric and knob-repeat DNA, and suppresses the transcription of small DNA repeats in developing MMCs. Strikingly, in contrast with its meiosis-specific function, AGO104 specifically accumulates in nucellar cells surrounding the developing MMCs, indicating that it regulates indirectly meiosis initiation by the production of a mobile signal (e.g. small interfering RNA (siRNA)) rather than by a direct cell-autonomous control mechanism (Fig. 3; Singh *et al*., 2011). AGO104 is therefore considered to be a major component of a conserved small RNA machinery that regulates the mitotic-to-meiotic switch in a noncell-autonomous manner.

Similarly, in mouse, one specific member of the ARGONAUTE family, for example AGO4, regulates the mitosis-to-meiosis transition. However, in contrast with maize AGO104, loss of AGO4 causes a premature entry into meiosis and leads to epigenetic changes (reduced silencing of sex chromosomes and reduced micro-RNA (miRNA) levels), inducing apoptotic cell death (Modzelewski *et al*., 2012). This is in line with studies in *Caenorhabditis elegans*, in which two ARGONAUTES, for example ALG-1 and ALG-2, are required for germ cell proliferation and entry into meiosis (Kimble & Crittenden, 2007; Bukhari *et al*., 2012). Thus, also in metazoans, the mitotic-to-meiotic switch is controlled by a noncell-autonomous miRNA mechanism (Kimble, 2011).

Several studies in metazoans have demonstrated that germline-specific PIWI-like proteins and associated small RNAs (PIWI-interacting RNAs (piRNAs)) are essential in the differentiation and maintenance of the germline (Cox *et al*., 1998, 2000; Carmell *et al*., 2002; Kuramochi-Miyagawa *et al*., 2004; Thomson & Lin, 2009; Unhavaithaya *et al*., 2009). Moreover, the loss of PIWI-like proteins causes an arrest of the meiotic cell cycle at prophase I (Houwing *et al*., 2008; Beyret & Lin, 2011), similar to that observed for several AGOs (Nonomura *et al*., 2007), indicating that, in both plants and animals, entry into meiosis is regulated by PIWI-piRNA-associated epigenetic programming and post-transcriptional gene regulation (Juliano *et al*., 2011). However, as the underlying molecular mechanism (e.g. RNA targets, transposable element (TE) activation or other epigenetic modifications) is as yet unknown (Holmes & Cohen, 2007), more studies are needed to elucidate the exact process by which AGOs and PIWIs regulate the mitosis-to-meiosis switch, and to what extent they control apomictic reproduction in plants (Grimanelli, 2012).

### 4. Molecular regulation of (post-)meiotic cell plate formation

Alterations in post-meiotic cytokinesis are an important route for 2n gamete formation, and several proteins involved have been characterized. In Arabidopsis, one of the major regulators is TES (TETRASPORE), a kinesin that shares homology with tobacco NACK (NPK1-activating kinesin-like) proteins (Yang *et al*., 2003; Tanaka *et al*., 2004). NACK1 and NACK2 encode members of the kinesin superfamily and regulate *de novo* cell plate formation by activation of a mitogen-activated protein kinase (MAPK) signaling cascade. In tobacco, this MAPK module includes NPK1, NQK1 and NRK1/NTF6, and activation of this signaling pathway is triggered by the binding of NACK proteins to NPK1 in late M-phase (Nishihama *et al*., 2001; Jonak *et al*., 2002; Soyano *et al*., 2003; Takahashi *et al*., 2004). Finally, at the end of the cascade, NRK1/NTF6 phosphorylates the MT-associated protein MAP65-1, stimulating phragmoplast expansion (Takahashi *et al*., 2010). Deficiency of NACK1, or its Arabidopsis homolog HINKEL (HIK), or any downstream MAPK component, largely inhibits somatic cell plate formation and induces severe defects in plant growth and development (Nishihama *et al*., 2002; Strompen *et al*., 2002).

Strikingly, unlike NACK1/HIK, loss of TES does not induce any defect in somatic cytokinesis, but instead leads to a complete failure of male meiotic cell plate formation (Hulskamp *et al*., 1997; Spielman *et al*., 1997). Cytological studies have demonstrated that the RMA, for example the MT structure that mediates vesicle deposition and cell plate formation, in *tes* PMCs is highly disorganized or absent, leading to a complete loss of meiotic cytokinesis (Yang *et al*., 2003). As a result, *tes* PMCs produce large spores containing all products of a single meiosis in one cytoplasm (Spielman *et al*., 1997; Tanaka *et al*., 2004). As the resulting ‘tetraspores’ show (partial) nuclear fusion before pollen mitosis I (PMI), subsequent spore development generates tricellular pollen grains that contain either multiple haploid/diploid or two tetraploid sperms (Spielman *et al*., 1997). In line with this, *tes* generates polyploid progeny together with a small set of diploid offspring (Scott *et al*., 1998).

Similar to the NACK-PQR pathway in tobacco, regulation of cytokinesis by TES or its somatic homolog HIK in Arabidopsis also involves a MAPK signaling cascade, including three MAPKKKs (ANP1, ANP2 and ANP3; Krysan *et al*., 2002), the MAPKK MKK6/ANQ1 and the MAPK MPK4 (Takahashi *et al*., 2010). As MPK4 phosphorylates a subset of MAP65 proteins (AtMAP65-1, AtMAP65-2 and AtMAP65-3), for example MT-associated proteins that mediate the structural organization of the phragmoplast (Muller *et al*., 2004; Gaillard *et al*., 2008; Farquharson, 2011; Ho *et al*., 2011), the regulation of meiotic cell plate formation by TES is thought to be controlled by a MAPK-mediated activation of MAP proteins (Sasabe *et al*., 2011). In line with this, protein interaction assays revealed that TES, ANP3, MKK6 and MPK4 indeed constitute a MAPK signaling cascade (Zeng *et al*., 2011), and cytological analysis has demonstrated that functional loss of MPK4 and MKK6/ANQ1 also induces defects in meiotic cell plate formation (Soyano *et al*., 2003; Kosetsu *et al*., 2010), yielding multinuclear spores that develop into diploid or polyploid pollen (Zeng *et al*., 2011).

### 5. Molecular factors controlling pre-meiotic ploidy stability

Although 2n spore formation through pre-meiotic genome doubling is rather rare in plants, recent studies have revealed a genetic background for this phenomenon. To ensure the haploid gametophytic genome content, sexually reproducing organisms need to control the ploidy level of meiocyte initials by maintaining them at the diploid state. Several proteins maintaining somatic ploidy integrity have been identified (Schnittger *et al*., 2002; Boudolf *et al*., 2004; Imai *et al*., 2006; Larson-Rabin *et al*., 2009; Iwata *et al*., 2011); however, these reports focus on vegetative tissue and do not include the analysis of meiotic precursor cells. So far, only two proteins have been found to be involved in meiotic ploidy control: the 40S ribosomal protein (rp) S6 kinases S6K1 and S6K2.

Functional loss of S6K in *s6k1s6k2*/++ and *S6K* RNAi Arabidopsis typically results in PMCs containing 20 chromosomes instead of the 10 in diploid PMCs. The occurrence of tetraploid PMCs, together with an endoploidy increase in *s6k* petals and leaves, indicates that S6K plays an essential role in the ploidy maintenance of vegetative and pre-meiotic cell lineages. Moreover, ectopically polyploidized *s6k* cells have a duplicated centromere number, indicating that they originate from endomitosis rather than from endoreduplication (Henriques *et al*., 2010).

S6K plays an important role in plant growth and development by modulating translational capacity through the phosphorylation of rpS6, a critical component of the 40S ribosomal subunit (Dufner & Thomas, 1999; Meyuhas, 2008). By contrast, Henriques *et al*. (2010) found that S6K controls the somatic endoploidy level by repressing the transcriptional activation of major cell cycle genes, including *CDKB1;1* and *CDKA*. Moreover, as S6K mediates the nuclear localization of RBR (RetinoBlastoma Related) and RBR depletion induces ectopic polyploidization through excessive release of E2Fs, *s6k* endopolyploidy most probably results from an enhanced cell proliferation caused by the mislocalization of RBR (Fig. 3). This hypothesis is supported by the observation that RBR binds to E2FA and forms a repressor complex that regulates proper cell cycle progression and inhibits ectopic polyploidization (Magyar *et al*., 2012).

Another example in which polyploid gametes are formed by the modulation of cell cycle-related genes has been described by Li *et al*. (2009). By ectopically expressing *FZR2*/*CCS52A1* in flowers, these authors not only induced endomitosis in developing petals, but also generated polyploid pollen. The WD-40 repeat-containing protein FZR2/CCS52A1 is a substrate-specific APC/C activator, promoting the transition of the mitotic cell cycle into endoreduplication (Vanstraelen *et al*., 2009). Accordingly, loss and overexpression of FZR2/CCS52A1 severely reduces and enhances endoreduplication, respectively (Larson-Rabin *et al*., 2009). In line with this, ectopic *FZR2*/*CCS52A1* expression, driven by the flower-specific *APETALA3* (*AP3*) promoter, induces polyploidy in several flower tissues, including male gametes (Li *et al*., 2009). However, because of the lack of cytological examination, it is unclear whether these polyploid spores result from pre- or post-meiotic genome duplication.

### 6. Other potential regulators of 2n gamete formation

A characteristic phenomenon inherent to functional 2n gametes in diploid plants is the production of triploid offspring. Indeed, although polyploid seeds can originate from a multitude of defects, triploid seeds can only result from diplogametes in the parental line. Based on this correlation, WYRD and RBR were found to play a role in gametophytic ploidy control.

In a recent report, Kirioukhova *et al*. (2011) demonstrated that Arabidopsis *wyr-1*/+ produces triploid offspring. WYR encodes a putative ortholog of the highly conserved inner centromeric protein INCENP, and contains a characteristic C-terminal domain, a coiled-coil domain and an IN-box aurora B-binding domain with four amino acid residues that are conserved form yeast to mammals. In general, INCENP functions in a complex with aurora kinases, survivin and borealin (chromosome passenger complex, CPC) to regulate different aspects of the cell cycle; including chromosome segregation, the spindle assembly checkpoint and cytokinesis. Hence, triploid *wyr-1*/+ progeny could result from 2n gametes that are formed by a failure of CPC-dependent chromosomal segregation during male or female gametogenesis. In support of this, ectopic genome duplication, through defects in CPC, has already been observed in somatic tissue (Nguyen & Ravid, 2006). By contrast, the formation of dyads and triads in *wyr-1*/*+* male sporogenesis indicates that 2n gametes are formed through meiotic nonreduction. Functional analysis of CPC in meiosis is impeded by the embryonic lethality of corresponding null alleles. However, weak alleles, RNAi and chemical inhibition have demonstrated that CPC plays an important role in different steps of meiosis, including chromosome condensation and alignment, spindle assembly and cytokinesis (Gao *et al*., 2008; Resnick *et al*., 2009). Although Aurora-B is the main CPC aurora component, genetic studies have revealed that it is substituted by Aurora-C in meiotic cells (Li *et al*., 2004; Fernandez-Miranda *et al*., 2011; Santos *et al*., 2011). Moreover, similar to WYR in Arabidopsis, loss of Aurora-C function induces meiotic restitution in mouse oocytes and human spermatozoa (Dieterich *et al*., 2007, 2009; Yang *et al*., 2010). In plants, however, little is yet known about the role of WYR/INCENP and other CPC components in meiosis and 2n gamete formation.

Functional loss of RBR in Arabidopsis *rbr*/+ also yields triploid progeny. RBR is the single Arabidopsis ortholog of the highly conserved animal tumor repressor protein pRB, a key regulator of the mitotic cell cycle. More specifically, pRB forms a complex with E2F and negatively regulates mitotic progression by repressing the G1-to-S transition (Weinberg, 1995; Zhang *et al*., 1999). RBR is essential for plant development, as it plays a role in cell proliferation and differentiation, stem cell maintenance and organ production (Borghi *et al*., 2010; Gutzat *et al*., 2012). Moreover, recent reports have revealed that RBR is also necessary for the differentiation and cell fate establishment of gametophytic organs (Ebel *et al*., 2004). Loss of RBR in micro- and megagametogenesis induces excessive proliferation and polyploidization, yielding spores that contain extranumerary haploid and polyploid nuclei (Johnston *et al*., 2008, 2010; Chen *et al*., 2009). Hence, triploid *rbr*/+ offspring most presumably result from 2n pollen or eggs that originate from a post-meiotic genome duplication event. Moreover, as the gametophytically lethal *rbr* allele is not transmitted by the female, Johnston *et al*. (2010) have suggested that triploids result from the fusion of a haploid egg with a diploid *rbr* sperm.

## V. Conclusions and future directions

Haploid spore formation is a complex process that is tightly regulated by a large network of molecular factors. Aberration of this process may lead to diploid gametes and may consequently confer sexual polyploidization. From an evolutionary perspective, this pathway most probably constitutes the predominant route for polyploid establishment. However, to fully elucidate the contribution of 2n gametes in ancient polyploidization, future research should focus on the unraveling of polyploid origins and the induction of sexual polyploidization, for example through abiotic stress or spontaneous mutations. In addition, further elucidation of the genetic and molecular processes underlying 2n gamete formation may enhance our understanding of plant reproduction, and provide new cues for the generation of polyploid crops and hybrids and the implementation of innovative breeding strategies (e.g. apomixis, reverse breeding). As the physical isolation of reproductive tissues is now technically feasible (micro-dissection), genome-wide analysis techniques, such as micro-arrays and RNA sequencing, may provide additional insights into the regulatory mechanisms of 2n gamete formation. Moreover, further improvement of cytological tools to detect and examine cellular aspects of meiotic restitution and 2n gamete formation may also contribute substantially to a full understanding of the stability of plant reproductive ploidy. Innovative techniques, such as live imaging of meiosis and recombinant chromosome markers, may thereby play an important role. Moreover, with the emerging importance of epigenetics in reproductive specification and maintenance, we also believe that future studies on meiotic chromatin dynamics, DNA methylation and RNA processing may provide essential insights into the molecular control of 2n gamete formation and sexual polyploidization.
